# Racial humility over competence: Addressing anti-Black racism and healthcare leadership responsibility

**DOI:** 10.1177/08404704231186807

**Published:** 2023-07-06

**Authors:** Notisha Massaquoi

**Affiliations:** 1 33530 University of Toronto, Scarborough, Ontario, Canada.

## Abstract

Health leaders’ response to anti-Black racism should not solely be a reaction to the police brutality and violence faced by Black communities. As part of healthcare leadership practice, we are responsible for recognizing the profound impact of anti-Black racism on all aspects of society, organizations, policies, practices, and behaviours. Based on interviews with health leaders responsible for implementing anti-Black racism strategies in their organizations, racial humility has been proposed as a necessary skill required to dismantle anti-Black racism. This requires a non-negotiable commitment, evaluation, and assessment of accountability, as well as the power to disrupt the impact of historical inequities, disparities, and discrimination experienced by Black community members. Racial humility is perceived as creating an ongoing practice to address anti-Black racism in healthcare, moving leaders from competence and discussion to reflection and transformative action.

## Introduction

The public murder of George Floyd at the hands of police officers was 8 minutes and 46 seconds on constant replay over television and social media. This was a turning point in awakening many health leaders in Canada to the realities of anti-Black racism and the pervasiveness of the spectacle of the Black death. For some, it was also a realization that Black community members had been subjected to years of continuous broadcast of public murders without public outcry from non-Black members of society. Notably, George Floyd’s murder took place amidst the backdrop of the world reeling from the worst global pandemic in over a century, just as reports began emerging about the disparately negative impact of COVID-19 on Black communities in Canada. Desegregated race-based COVID-19 data highlighted the persistent structural inequities in healthcare, income, education, access to government resources, and the impact of colour-blind policies.^1,7^ Canada’s largest city saw 83% of COVID-19 cases being attributed to racialized community members, with the Black community carrying the burden of the highest rates at 21%, despite only making up 9% of the population.^2^

Canadian healthcare organizations experienced heightened scrutiny due to the lack of Black representation among staff, clinicians, and administrators; the limited health programs targeting Black communities; and the lack of Black student mentorship and internship opportunities. The absence of race-based data collection to measure and monitor health disparities for Black community members in terms of access, disease prevalence, and treatment within the healthcare system has been an ongoing struggle.^8-10^ Healthcare governance has also been called into question with the revelation that only 1.9% of hospital board members nationally identified as Black, providing solid evidence of the lack of Black input in decision-making at the highest levels.^3^

During this racial awakening, health leaders with limited guidance or expertise were called upon to lead and address how the Canadian healthcare system is complicit in systemic anti-Black racism. Under the public pressure for racial justice, a flurry of statements condemning anti-Black racism and police brutality appeared in employee listservs, newsletters, press releases, and corporate web sites. Healthcare sector Chief Executive Officers (CEOs) went to great lengths to assure staff, patients, and the public of their commitment to anti-Black racism and the safety and well-being of all members of their communities. However, as fast as these statements to the community arose, critiques appeared just as quickly by those who perceived the statements as performative acts, amounting to nothing more than empty platitudes.

Statements in response to the killing of George Floyd reflect an unholy alchemy of risk management, legal liability, and trustee anxiety as opposed to strategies and tactics for institutional transformation grounded in an acknowledgement of anti-Black racism and White supremacy.^5^

After these statements were released, health leaders found themselves needing guidance on how to start fixing this problem and, most importantly, how to lead this change. It was evident that there was a dearth of research exploring the best organizational practices or the experiences of Canadian health leaders implementing anti-Black racism strategies. Drawing from the literature and consultations with senior health leaders, this article explores the core skills, knowledge, and attributes one needs to contemplate before beginning to address the elimination of anti-Black racism in Canadian healthcare organizations and institutions.

For the most part, the health leaders who were interviewed had already embraced diversity, inclusion, and equity concepts, but explicit strategies to address and eliminate anti-Black racism were a new undertaking for most. This work required leaders to prioritize the Black members of their communities and to listen to, be advised by, and cater to the needs of Black people.^11^ This has not been a position that health leaders in a Canadian context have been comfortably placed in. It was easier to subsume Black community priorities into a single group with all racialized communities or those who fall under the BIPOC^1^ umbrella, which was growing in popularity. Solely looking at the needs of Black communities was often seen as challenging to explain to key stakeholders, particularly senior leadership teams, boards of directors, and donors. Health leaders made public commitments to stand in solidarity with Black communities and support their fight for justice by releasing lists of what their organizations were going to do or hoped to do. However, very few outlined concrete plans that included specific actions that the leaders themselves would undertake to permanently eliminate anti-Black racism from healthcare organizations and the healthcare sector as a whole.

The challenge of leading an organization that is eliminating anti-Black racism is that one is required to create a culture that allows for transparent acknowledgement that anti-Black racism exists in the organization. It also requires documentation of the magnitude of the proliferation of anti-Black racism throughout the entire organization. All organizational members must be interrogated to secure further recognition of anti-Black racism and to determine how it has created a system of disproportionate opportunity and penalties based solely on race.^11^ In order to truly engage in the work to eliminate anti-Black racism and live up to the public statements about commitment to this activity, health leaders must ensure that they and their organization are held accountable by the Black communities they serve. They must also develop a deep understanding of systemic anti-Black racism within their organization, the healthcare system, and society as a whole, and must commit to securing the survival and well-being of all Black people they serve and employ.^12^ We cannot hope to eliminate anti-Black racism with a list of to-dos. Instead, health leaders need to ensure that they are working toward the elimination of anti-Black racism through a lifelong, ongoing, and dynamic process. As part of that process, they must genuinely commit to engaging with and listening to the Black community members who are most impacted by anti-Black racism, as well as acquiring the education required to make informed decisions that will have lasting effects. This requires an accelerated process of examining how power, inequality, and anti-Black racism play out at every level of your organization. These are not one-time activities; they must be consistently repeated throughout your career, not only in response to public crisis.

## Accountability requires racial humility

Health leaders have discussed turning to anti-Black racism training or workshops as the first activity in the quest to engage in the elimination of anti-Black racism. There was a natural inclination to want to quickly improve ones’ understanding of anti-Black racism in order to maintain leadership status. Competence and mastery were often preferred when beginning the leadership journey to eliminate anti-Black racism. Leaders often assumed that they could learn a quantifiable set of attitudes and skills that would allow them to work effectively within the racial context of Black community members. Although there was a strong focus on knowledge acquisition, there was limited engagement with social justice activities. Furthermore, many focused on understanding anti-Black racism as a technical and communication skill to learn how to say the right thing and when to say it. This orientation of non-Black leaders seeking to learn about the history of anti-Black racism and how it functions systemically is often seen as sufficient, with little need to strive for or engage in social justice activities to eliminate oppression.^13^

However, the development of racial humility as a leadership quality has proven to be more effective and long-lasting. The concept of humility was recognized as an effective tool for training physicians to deliver culturally appropriate medical care, and its origins can be attributed to Tervalon and Murray-Garcia.^14^ It entails a lifelong commitment to self-reflection and analysis to address the power imbalance in the physician–patient relationship and develop mutually beneficial, non-paternalistic advocacy partnerships with individuals and communities.^4^ This model emphasizes self-critique and respect between healthcare providers and clients, which is crucial to improving health outcomes for oppressed populations.^15^ Practitioners who use a humility model are expected to challenge the institutional forces and processes that shape the practitioner–client relationship.^6^

The racial humility concept, as proposed in this research, offers a solid foundation to begin the work of eliminating anti-Black racism compared to other models that promote gaining competence or mastery before beginning this work. Expanding on this original understanding of humility to build cultural competence in healthcare, racial humility requires a fundamental change in how leaders think about, understand, and interact with the world around them to include Black community members in the most meaningful and positive way. Racial humility advocates for lifelong engagement in addressing anti-Black racism versus a discrete endpoint obtained with training or workshops. Racial humility requires leaders to account for the structural forces that limit Black individuals’ experiences and opportunities within their organizations, and challenges leaders to address racial inequalities at individual and institutional levels.^16^ Non-Black leaders are not simply creating environments where they feel more comfortable with Black community members; they are focusing on being self-aware of the anti-Black biases and assumptions they may bring when working with Black colleagues or patients. Racial humility supports the recognition that perpetuation of marginalization and discrimination against Black community members are among the deeply ingrained values of an organization that has not eliminated anti-Black racism.^17^

## Racially humble leaders and racially responsive organizations

As health leaders begin addressing the often invisible and covert nature of anti-Black racism, the goal is to create racially responsive organizations that build strong organizational policies, practices, and structures designed to eliminate anti-Black racism, inequity, and exclusion. It is not about teaching or training people to engage with Black community members in good and moral ways. It is about requiring organizations to examine policies, procedures, unspoken norms, and routines that have created barriers and, oftentimes, traumatic experiences for Black staff and patients. It is about digging through every aspect of an organization to learn about whom it was initially built for, who was and is still being excluded, and who is being harmed, and then developing a plan of correction. It is about reviewing everything that makes an organization what it is and understanding who is a part of the organizational community. This requires learning about demographic data through a race-based lens, such as who is being hired, fired, or promoted and who is quitting, and ensuring Black community members experience no specific negative patterns.^17^

Non-Black leaders are required to look at who makes up the top of their organization and who makes up the bottom when considering anti-Black racism. In the Canadian healthcare system, we have become complacent, as shown in the lack of Black leadership and limited progression of Black employees in organizations through promotion. Research shows that Black employees face more significant obstacles to advancement than other racialized groups; are less likely than their White peers to be hired, mentored, and promoted; and encounter lived experience at work that are demonstrably worse than other racialized groups.^18^ Leaders consulted in this process overwhelmingly noted a gap between what their organizations traditionally had been saying versus what they had been doing to promote diversity and inclusion. The espoused addressing of anti-Black racism and the reality of Black staff outcomes appear misaligned when one can visually see the lack of Black senior leaders in their organizations and the overrepresentation of Black employees in the lowest-paid positions in the organization. If health leaders want to walk the talk, they must spearhead sustainable Black community-focused interventions ranging from entry-level recruitment to CEO succession. Many experts have suggested that the racially humble leader must be committed to creating a more equitable and inclusive workplace that focuses on attracting Black talent and nurturing those employees throughout their careers to access leadership opportunities.

Mapping the journey travelled through an organization as a Black employee or a patient is a crucial exercise in understanding how anti-Black racism works in the environment you lead and what needs to change. The critical work and assessment that the racially humble leader is committing to undertake will examine where anti-Black racism exists from an employee perspective, including recruitment, retention, performance recognition, and promotions. It will also look at where Black community members are located in the organization and which teams or departments they are overrepresented versus underrepresented or non-existent. The aim of this process is to understand what Black community members are experiencing in your organization as both employees and recipients of your services. This can only be achieved by consulting extensively with Black community members and partnering with healthcare organizations that serve Black communities well.

Racial humility places the health leader in a perpetual learning mode that requires relinquishing power, control, and authority.^19^ It is common for non-Black leaders to become overwhelmed with the amount of learning and unlearning required to address anti-Black racism, with some initially choosing to delegate the work to a single individual in the organization or to EDI offices. When non-Black leaders abdicate their responsibility to address anti-Black racism within their organizations, the few Black leaders often begin to feel that they are expected to become “cultural ambassadors” who must not only address the needs of all Black employees, but act as educators and interpreters for non-Black senior leaders in the organization. They are essentially left to do two jobs: the official one they were hired to do, and the expected one that requires them to act as a champion for members of the Black community. The research noted the issue of “diversity fatigue” arising from constantly engaging in task forces, training, and racial conversations.^20^ Black leaders were often asked to take the lead on this work, whereas non-Black leaders could choose to sit out or join in, depending on whether they felt ready or competent and whether they wanted to. Racial humility raises leaders’ awareness and sensitivity to the burden of addressing anti-Black racism that needs to be evenly distributed across the organization, with non-Black leaders with the highest authority carrying the heaviest burden.

Shifting from a sense of mastery to accountability requires asking challenging questions to move toward more racially responsible work. Mastery asks individuals to ‘understand’ others, whereas accountability requires action and results. The way we motivate individuals and organizations to ask the right questions and act on anti-Black racism is a critical step in the learning process. As such, we must ask questions that move us beyond simplistic and reductionist conversations about difference and ask more questions about our institutions and ourselves. This includes posing a series of questions essential for self-reflection at the individual and institutional levels (Table 1). These questions can evaluate an institution’s effectiveness and can guide staff training, professional development, and strategic planning.

**Table 1.** Anti-Black racism reflection and accountability questions.

Goal			
Organizations, who hold themselves accountable to Black communities, immerse themselves in understanding systemic anti-Black racism and commit themselves to ensuring the survival and well-being of Black people will be the most successful in their work with and for Black communities.			
Self-reflection questions			
What is my personal philosophy when addressing anti-Black racism?	What are the risks I am willing to take to address anti-Black racism? Where and when will I need to show courage?	How will I be held accountable for my work in addressing anti-Black racism?	How will the Black community see my work? What changes should they expect?
Individual accountability questions			
Do I understand how our mission, vision, and values are being experienced by Black communities?	How do I ensure that our anti-Black racism plan ties directly to our strategic plan?	How am I addressing power imbalances and resistance to eliminate anti-Black racism in our organization?	How do I publicly present and share our work to address anti-Black racism?
Organizational accountability questions			
What recruitment, retention, and advancement practices are we implementing to increase Black community participation in our organization at all levels?	How are we developing senior leadership and governance skill levels to address anti-Black racism?	What policies need to be developed and implemented to address anti-Black racism? Have we conducted a full equity audit of all policies to address any systemic barriers created by policies and practices?	What is our plan to engage in effective mandatory anti-Black racism education and training throughout the entire organization?
How does my organization support social action to address anti-Black racism?	How does my organization engage in bias-free race-based data collection?	What is my organization’s public presentation and commitment to address anti-Black racism?	What is our Black community engagement and partnership building plan?

## Conclusion

This article introduces racial humility and highlights the intersections of individual and organizational accountability. A racially humble leader is defined as one who pursues a transformative agenda to challenge the status quo. Racial humility requires health leaders to confront imbalances rather than simply acknowledge they exist. The healthcare system must prioritize the needs of the most vulnerable, beginning with developing a framework that confronts the systemic forces that drive the health, economic, and social inequalities that marginalize people on both the individual and institutional levels. Racial humility offers a personal and organizational model to address these systemic inequalities. Competence models have provided a springboard for conversations about what it means to understand anti-Black racism, but have not produced transformative agendas to address such inequalities. Focusing on competence to address inequality is no longer sufficient if our healthcare system wants to engage, lead, and ultimately change outcomes for Black communities. Racial humility challenges us to ask difficult questions instead of reducing Black community members to experiences we have learned about from anti-Black racism training. Asking critical questions, such as those presented in this article, will challenge our leadership practices, transform our organizations, and provide a more profound approach to implementing the required individual and organizational change that will lead to effective long-term practice of eliminating anti-Black racism.

## Note

1. An acronym for Black, Indigenous, and People of Colour that is used in the United States. It is intended to centre the experiences of Black and Indigenous groups and to demonstrate solidarity between communities of colour.
